# A low-tech method for monitoring survival and growth of coral transplants at a boutique restoration site

**DOI:** 10.7717/peerj.15062

**Published:** 2023-05-24

**Authors:** Sarah Frias-Torres, Claude Reveret, Kerstin Henri, Nirmal Shah, Phanor Hernando Montoya Maya

**Affiliations:** 1 Nature Seychelles, Island Conservation Centre, Praslin, Republic of Seychelles; 2 Smithsonian Marine Station, Fort Pierce, FL, USA; 3 CREOCEAN, La Rochelle, France; 4 Corales de Paz, Santiago de Cali, Colombia

**Keywords:** Coral gardening, Coral transplants, Indian Ocean, Seychelles, Restoration

## Abstract

**Background:**

Coral reef restoration projects are becoming a popular corporate environmental responsibility activity at hotel resorts. Such involvement of private businesses offers the potential to expand restoration into a new socioeconomic sector. However, the scarcity of user-friendly monitoring methods for hotel staff, but robust enough to detect changes over time, hinders the ability to quantify the success or failure of the restoration activity. Here, we present a monitoring method of easy application by hotel staff, without scientific training, using the standard resources available at a hotel resort.

**Methods:**

Survival and growth of coral transplants were evaluated over 1 year at a boutique coral reef restoration site. The restoration was tailored to the needs of a hotel resort in Seychelles, Indian Ocean. A total of 2,015 nursery-grown corals of branching (four genera, 15 species), massive (16 genera, 23 species), and encrusting (seven genera, seven species) growth types were transplanted to a 1–3 m deep degraded patch reef. A unique cement mix was used to transplant corals onto the hard substrate. On the north side of each coral selected for monitoring, we attached an 8.2 cm × 8.2 cm reflective tile. We used reflective tiles instead of numbered tags due to the expected amount of biofouling growing on the tag surface. Every coral was recorded with top view photography (perpendicular to the plane of coral attachment), with the reflective square in the field of view. We drafted a map of the site to facilitate navigation and re-sighting of the monitored colonies. Then, we developed a simple monitoring protocol for hotel staff. Using the map, and the reflective tiles, the divers located the coral colonies, recorded status (alive, dead, bleaching), and took a photograph. We measured the two-dimensional coral planar area and the change in colony size over time using contour tissue measurements of photographs.

**Results:**

The monitoring method was robust enough to detect the expected survival of coral transplants, with encrusting and massive corals outperforming branching corals. Survival of encrusting and massive corals was higher (50%–100%) than branching corals (16.6%–83.3%). The change in colony size was 10.1 cm^2^ ± 8.8 (SE). Branching coral survivors grew faster than massive/encrusting corals. A comprehensive approach to the boutique restoration monitoring experiment would have included comparisons with a control patch reef with a similar species composition to the coral transplants. However, the ability to monitor such a control site, in addition to the restoration site, was beyond the logistic capabilities of the hotel staff, and we were limited to monitoring survival and growth within the restoration site. We conclude that science-based boutique coral reef restoration, tailored to the needs of a hotel resort, combined with a simple monitoring method, can provide a framework for involving hotels as partners in coral reef restoration worldwide.

## Introduction

The prolonged worldwide decline of coral reefs (Hughes et al., 2018) requires the combination of traditional conservation with active restoration to support ecosystem recovery (Rinkevich, 2015). In the context of the United Nations Decade on Ecosystem Restoration, the goal is to prevent, halt and reverse the degradation of ecosystems worldwide. The private sector is identified as a key partner in the implementation of restoration initiatives, but a major barrier is the limited technical knowledge and capacity to implement such activities by staff working in this sector (UNEP, 2020).

Coral reef restoration projects are becoming a popular corporate environmental responsibility activity at hotel resorts. Hotel resorts show great potential to become a major contributor to coral reef restoration, beyond financial support, contributing anywhere from hands-on involvement of hotel staff to scalability and economic feasibility, as has been shown in case studies from Thailand and the Maldives (Hein, Couture & Scott, 2018) and by the Iberostar Hotels and Resorts at several locations in the Caribbean Sea, including Dominican Republic, Mexico, and Jamaica (Blanco-Pimentel et al., 2022). However, the scarcity of user-friendly monitoring methods for hotel staff, but robust enough to detect changes over time, hinders the ability to quantify the success or failure of the restoration activity. The private sector needs rapid and simple methods for monitoring coral reef restoration.

In Seychelles, Indian Ocean, engagement with the private sector for ecosystems and species restoration is well established. Private island owners participated in a restoration program to translocate globally threatened coastal birds and restore coastal biodiversity of global importance (Henri, Milne & Shah, 2004). The establishment of new populations of endangered species such as the Seychelles magpie robin *Copsychus sechellarum*, Seychelles warbler *Acrocephalus sechellensis*, and Seychelles fody *Foudia sechellarum* in restored habitats led to a downgrading of the threat status of these species and to enhancing eco-tourism potential. These results encouraged hotel owners to contribute to conservation efforts: Cousine, Fregate, and Denis islands are all funding their full-time conservation officers. The private islands, particularly Cousine, started full-scale conservation work of endemic birds and seabirds including research, monitoring, and new restoration projects (Shah, 2006).

In 1998, the coupling of El Niño and the Indian Ocean Dipole resulted in mass coral bleaching in the Indian Ocean (Spencer et al., 2000; Spalding & Jarvis, 2002). In the inner granitic islands of the Seychelles Plateau, live coral cover decreased to less than 3% in some areas (Graham et al., 2006). Since 1998, natural coral recovery has been extremely slow in Seychelles (Graham et al., 2006; Chong-Seng, Graham & Pratchett, 2014; Harris et al., 2014). In 2014–2015, as part of a large-scale coral reef restoration project in Seychelles (Frias-Torres & van de Geer, 2015; Frias-Torres et al., 2015; Frias-Torres, Montoya-Maya & Shah, 2019; Montoya-Maya et al., 2016), we completed a restoration experiment tailored to the specific needs of a hotel resort while complying with the science-based principles of ecological restoration (Suding et al., 2015). We define this type of client-targeted activity as boutique restoration.

Here, we present a low-tech method for monitoring the survival and growth of coral transplants. The method is of easy application by hotel resort staff, without scientific training, using the standard resources available at a hotel resort. The very shallow (1–3 m deep) degraded patch reef targeted for restoration was influenced by tides, ocean swells, and salinity fluctuations. In similar variable conditions, transplants of massive and encrusting corals show high survival rates up to 90% one-year post-transplantation (Coastal lagoons, Philippines, Gomez et al., 2014; coastal reef, Yemen; Seguin et al., 2008). We expected higher survival rates in massive and encrusting coral transplants when compared to branching coral transplants. Therefore, the monitoring method had to be robust enough to detect the different coral transplant survival rates.

## Materials & Methods

### Transplantation of nursery-grown corals

We implemented a formal arrangement through a Memorandum of Understanding (MOU) between Nature Seychelles and a five-star hotel resort under a donor-funded project. The purpose was to restore a patch reef tailored to the hotel resort’s needs (boutique restoration): accessible to hotel guests by snorkeling (1–3 m depth), and within 50 m from the beach (the length of an Olympic-sized swimming pool). The project complied with the four guiding principles of ecological restoration (Table 1).

**Table 1:** The four principles of ecological restoration. Application of the four guiding principles of ecological restoration (Suding et al., 2015) to the boutique coral reef restoration experiment.

Restoration principle	Application in this study
Increase ecological integrity	The complexity of coral assemblages was prioritized by outplanting 3 growth types (branching, massive, encrusting) and 45 species
Long-term sustainability	Restored coral reef consistent with very shallow water environmental context and designed to minimize human intervention in the long term.
Informed by the past and future	Historical knowledge of the existence of a diverse shallow coral reef at the restored site; outplanted corals grown from survivors of 1998 El Nino, potentially adapted for new warming events
Benefits and engages society	Capacity building by training hotel staff on monitoring survival and growth of outplanted corals. Production and screening of short videos explaining the coral reef restoration project and inviting tourists to snorkel the restored bay

**Figure 1:**
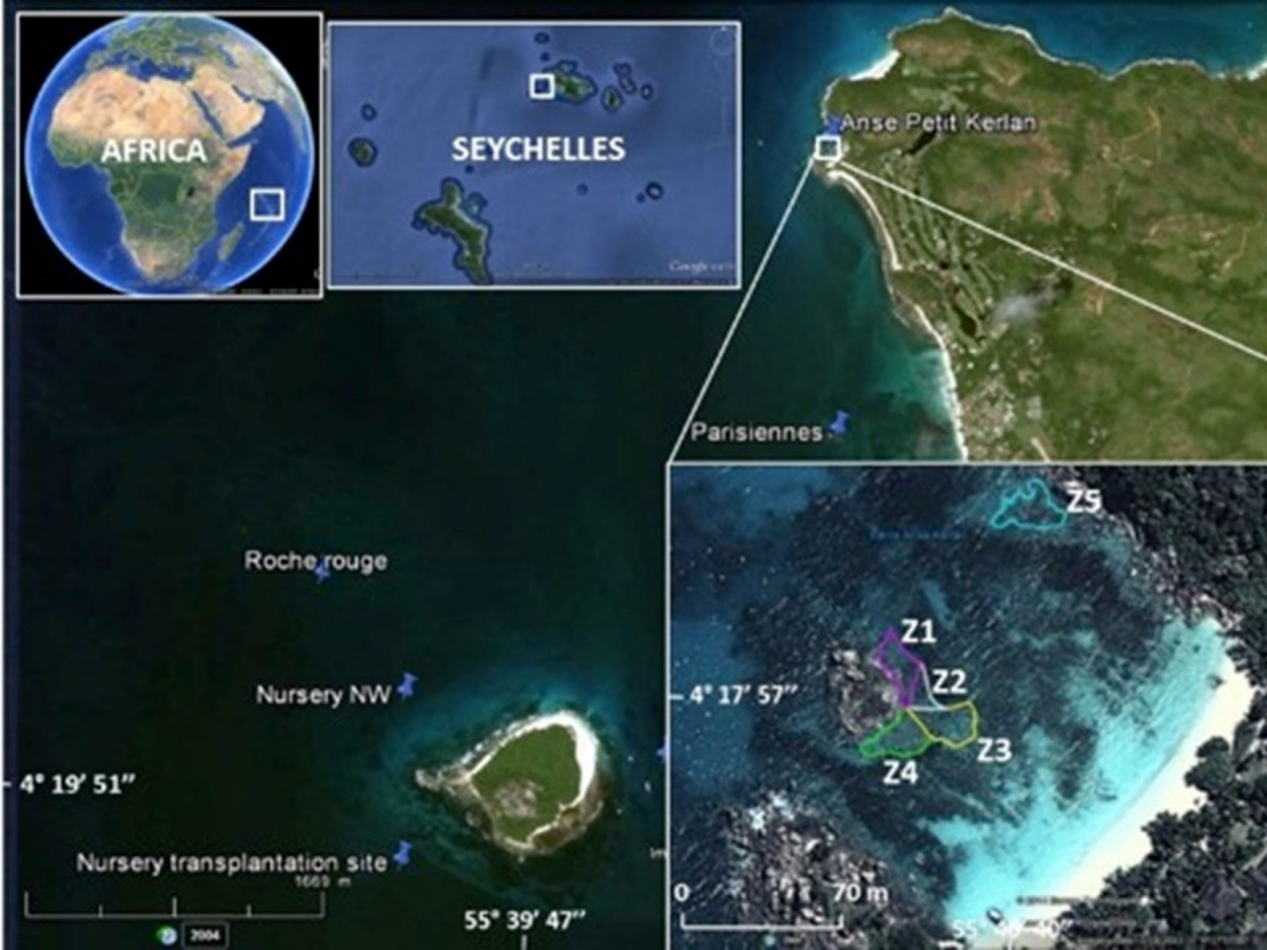
Study area. The map shows the location of the Cousin Island coral nursery, and donor sites (Les Parisiennes, Roche Rouge) in proximity to the transplantation area (Zone 1 through Zone 5) at Petite Anse Kerlan. Zone 5 was not included in this study. Image from Google Earth Pro © 2023 Maxar Technologies.

The boutique restoration experiment was conducted at Petite Anse Kerlan, Praslin Island (04°17′57.5″S, 055°40′40.0″E WGS84) (Fig. 1, Appendix S1: Methods). We used the coral gardening method (Rinkevich, 2006). The corals transplanted at this site were raised in midwater ocean nurseries (rope and net types) by fragmenting survivors of the 1998 mass bleaching event, found at two nearby patch reef donor sites (Les Parisiennes, Roche Rouge), and from corals of opportunity (rescued corals dislodged by storms or human activity). A description of the nursery site and nursery types is found elsewhere (Frias-Torres & van de Geer, 2015; Frias-Torres & van de Geer, 2015). Briefly, the rope and net midwater nurseries were floated 8 m below the sea surface. Each nursery was moored to angle bars hammered into the 17 m-deep sandy seabed. The midwater setup provided ideal growing conditions for the corals and minimized the danger of predatory snails and worms (Levy et al., 2010). We harvested corals from two rope nurseries, mainly *Acropora appressa*, and colonies derived from corals of opportunity (*Pocillopora* sp., *Acropora* sp.). Corals from the net nurseries were a mix of different growth types (Table 2), roughly 50% branching/tabular, 30% massive/sub-massive, and 20% encrusting.

**Table 2:** Coral species transplanted in the boutique restoration experiment. Names in bold were monitored as shown in Table S2. Scientific names of corals follow the World Register of Marine Species (WoRMS Editorial Board, 2016). Growth types are explained in Appendix S1. Credit for coral symbols: Woerner (2011).

Growth type	Species names
Branching /Tabular	
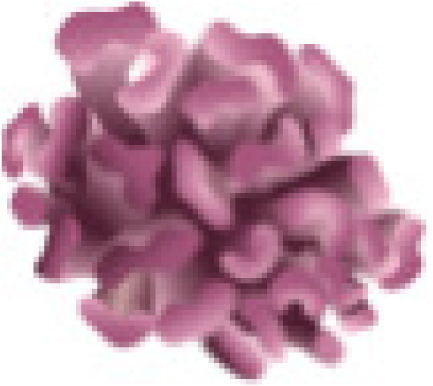	***Acropora abrotanoides*** *, A. appressa,* ***A. cytherea*** *,* ***A. humilis*** *, A. hyacinthus,* ***A. muricata*** *, A. vermiculata,* ***A. cf verweyi*** *,* ***Isopora brueggemanni*** *,* ***Pocillopora damicornis*** *,* ***P. grandis*** *, P. indiania,* ***P. verrucosa*** *, Stylophora pistillata, S. subseriata*
Massive /Submassive	
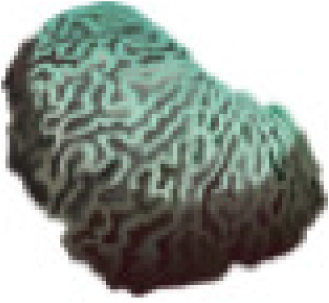	*Acanthastrea brevis, Astrea curta,* ***Astreopora myriophthalma*** *,* ***Coscinaraea monile*** *, Cyphastrea sp.,* ***Dipsastraea cf favus*** *, D. lizardensis,* ***Favites cf flexuosa*** *,* ***F. cf pentagona*** *, F. vasta,* ***Galaxea fascicularis*** *, Goniastrea edwardsi, Goniopora tenuidens,* ***G. pedunculata*** *,* ***Hydnophora exesa*** *, H. microconos, Lobophyllia hemprichii, Paramontastraea serageldini, Pavona decussata, P. explanulata, Platygyra acuta,* ***P. cf crosslandi*** *, Porites lobata*
Encrusting	
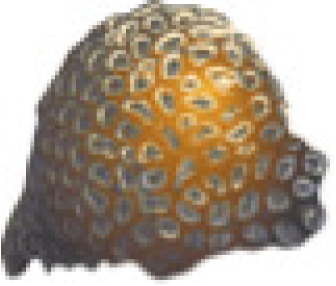	*Echinophyllia aspera,* ***Echinopora hirsutissima*** *, Favites pentagona, Leptastrea purpurea, Leptoseris incrustans, Psammocora haimiana, Turbinaria irregularis*

**Figure 2:**
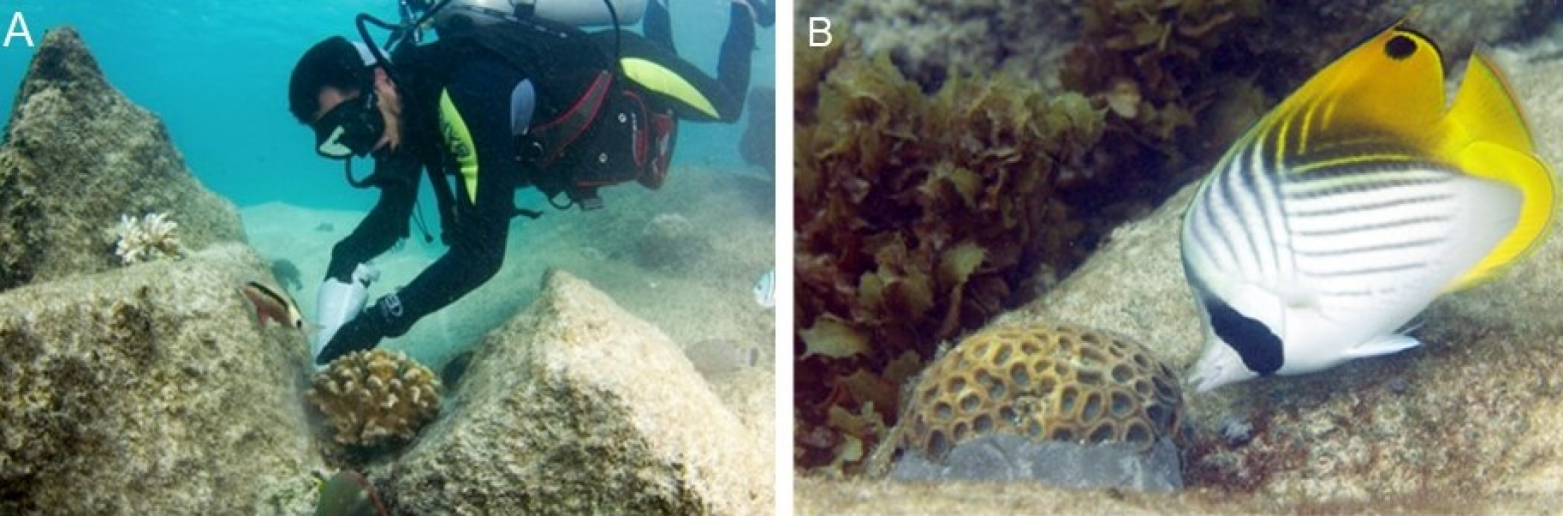
Coral cementing method. (A) The diver cements a nursery-grown coral using a chef pastry bag full of cement mix. (B) Side view of coral transplant showing cement at the base. Photo credit: N. Thake.

Coral transplantation at Petite Anse Kerlan focused on the 1–3 m deep degraded patch reef approximately 50 m offshore on the leeward side of a large rock island in the center of the bay. The study site was divided into five zones. Zones 1–4 radiated from the central large rock. Here, we transplanted branching, massive, and encrusting corals from the net nurseries. At Zone 5, located at the northeast side of the bay, we only transplanted branching corals from the rope nurseries (Fig. 1). In one month, a four-diver team transplanted 2,015 nursery-grown corals of branching (four genera, 15 species), massive (16 genera, 23 species), and encrusting (seven genera, seven species) growth types. We cemented corals onto the granite and limestone substrate with a unique mix developed by one of us (C. Reveret) (Fig. 2; Appendix S1 -Methods).

### Monitoring coral transplant survival and growth

We trained hotel staff to conduct a simple monitoring protocol every month to quantify the survival and growth of coral transplants based on location and growth type. We selected 48 corals for monitoring: 6 branching corals and 6 massive/encrusting corals per zones 1 through 4 (Table S1). Zone 5 was excluded from monitoring because it was more exposed and difficult to reach consistently by hotel staff. On the north side of each selected coral, we attached an 8.2 cm × 8.2 cm reflective tile. We used reflective tiles instead of numbered tags due to the expected amount of algal growth and biofouling growing on the tag surface. We drew a map of the site to facilitate navigation and re-sighting of the monitored colonies. Using the map, and the reflective tiles, the divers located the coral colonies, recorded status (alive, dead, bleaching), and took a photograph.

Survival status included detachment, bleaching, and disease. We measured the two-dimensional (2D) coral planar area using *in situ* underwater photographs. Every coral was recorded with top view photography (perpendicular to the plane the coral was attached to), with the reflective square in the field of view. Instead of using a monopod for camera support (Neal et al., 2015), the photographer stabilized the position by resting one finger on the substrate, effectively turning the arm into a monopod. This approach allowed for standardized analysis of measurements regardless of coral growth type.

### Image and statistical analysis

**Figure 3:**
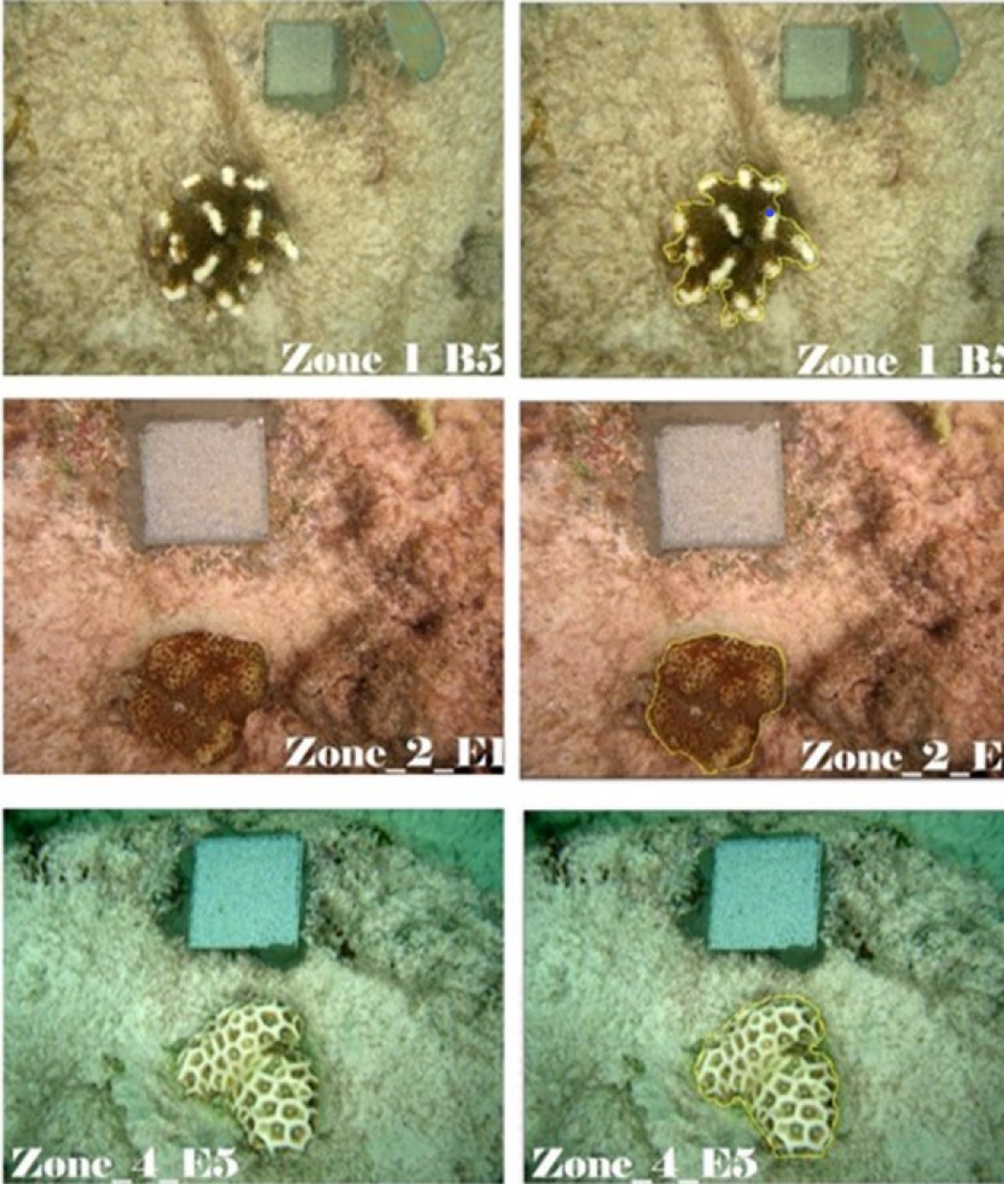
Measurement of live coral tissue using Image J. From top to bottom row, contour tracing for branching (Z1B5, *Pocillopora grandis*), encrusting (Z2E1, *Favites cf pentagona*), and massive (Z4E5, *Favites cf flexulosa*) corals. The perimeter of live coral tissue in the original photographs (left) is drawn as a yellow line (right) and Image J calculates the area delimited by the perimeter. Such an area was the coral planar area. Photo credit: N. Thake (original), P. Montoya-Maya (with Image J contour).

We measured the two-dimensional coral planar area and change in colony size over time using contour tissue measurements of photographs. Two experienced observers analyzed the coral photographs with the ImageJ software package (ImageJ, 1.48v; National Institutes of Health, Bethesda, MD, USA). Image J has been shown to facilitate rapid and precise measurements in coral monitoring (Neal et al., 2015). Each observer used the reflective square in the image to set the scale for each photograph and traced the contour of the live coral tissue (Fig. 3). To trace the contour of the coral, the first observer used a standard computer mouse and the second observer used a Wacom Intuos™ computer tablet with a stylus. All corals monitored were measured using both contouring methods. The calculated area based on the ImageJ algorithm was used as the live coral planar area. To evaluate the precision of the two independent observers, we calculated the variance for Observer as a random effect in the generalized linear mixed effect model (GLMM) used to explore the effects of zone and growth type on colony size.

High hotel staff turnover and lack of allocated time by hotel management prevented trained staff to implement monthly monitoring. Instead, we were limited to monitoring survival and growth between the end of the transplantation period (15 May 2014) to the end of the experiment period (24 April 2015).

Colony growth was determined as live coral planar area increment during the 11.5 months of the study. We compared coral planar area growth with annual extension rates published in the literature (Table S1). To convert the area increase we measured by photographic analysis to an annual extension rate in mm/y, we assimilated the coral planar area to a circle and calculated the increase in circle radius between the start and end of the observation period.

Due to the lack of monthly monitoring, we were unable to complete the survival analysis. Instead, we used a *t*-test to evaluate differences in survival related to size by comparing the planar area at transplant time of corals alive and dead at the end of the 11.5 months (345 days) experimental period. We used logistic regression analysis to quantify differences in survival related to growth type (branching *vs* massive/encrusting) and transplantation zone (zones 1 through 4). Here, growth type and transplantation zone were the predictor variables, and survival (number of corals alive at the end of the experimental period) was the dependent variable. Both *t*-test and logistic regression analyses were conducted in Statistica 6.0.

Colony growth was determined as a change in colony size (CCS) one year (11.5 months) after transplantation with zero (no growth), positive (growth), or negative values (loss of living tissue due to partial mortality or breakage). Data were tested for normality (Shapiro–Wilk test) and homogeneity of variance (Bartlett’s test). A sample *t*-test was used to test the significance of any change in colony size between the two sampling periods. To explore the effects of zone and growth type on CCS, we fitted a GLMM using a Gaussian error distribution with identity link function and zone and growth type as fixed factors with an interaction term. We used intercepts for individual colonies and observers as random effects. Visual inspection of residual plots did not reveal any obvious deviations from homoscedasticity (homogeneity of variances) or normality. Statistical significance (*P* < 0.05) was obtained by likelihood ratio tests of the full model with the effect against the model without the effect in question (Bolker et al., 2009). All statistical tests for CCS were done in R (R Core Team, 2013); for fitting GLMMs we used the lme4 (v1.1-6: Bates et al., 2014) package.

## Results

### Survival of coral transplants

We trained hotel staff to monitor coral transplants monthly, but the combination of a high staff turnover rate, staff job obligations not linked to coral monitoring, and rough weather conditions prevented consistent monthly monitoring as planned. Only the data we obtained at the start and end of the 11.5 month (345 days) experimental period were available for analysis.

**Figure 4:**
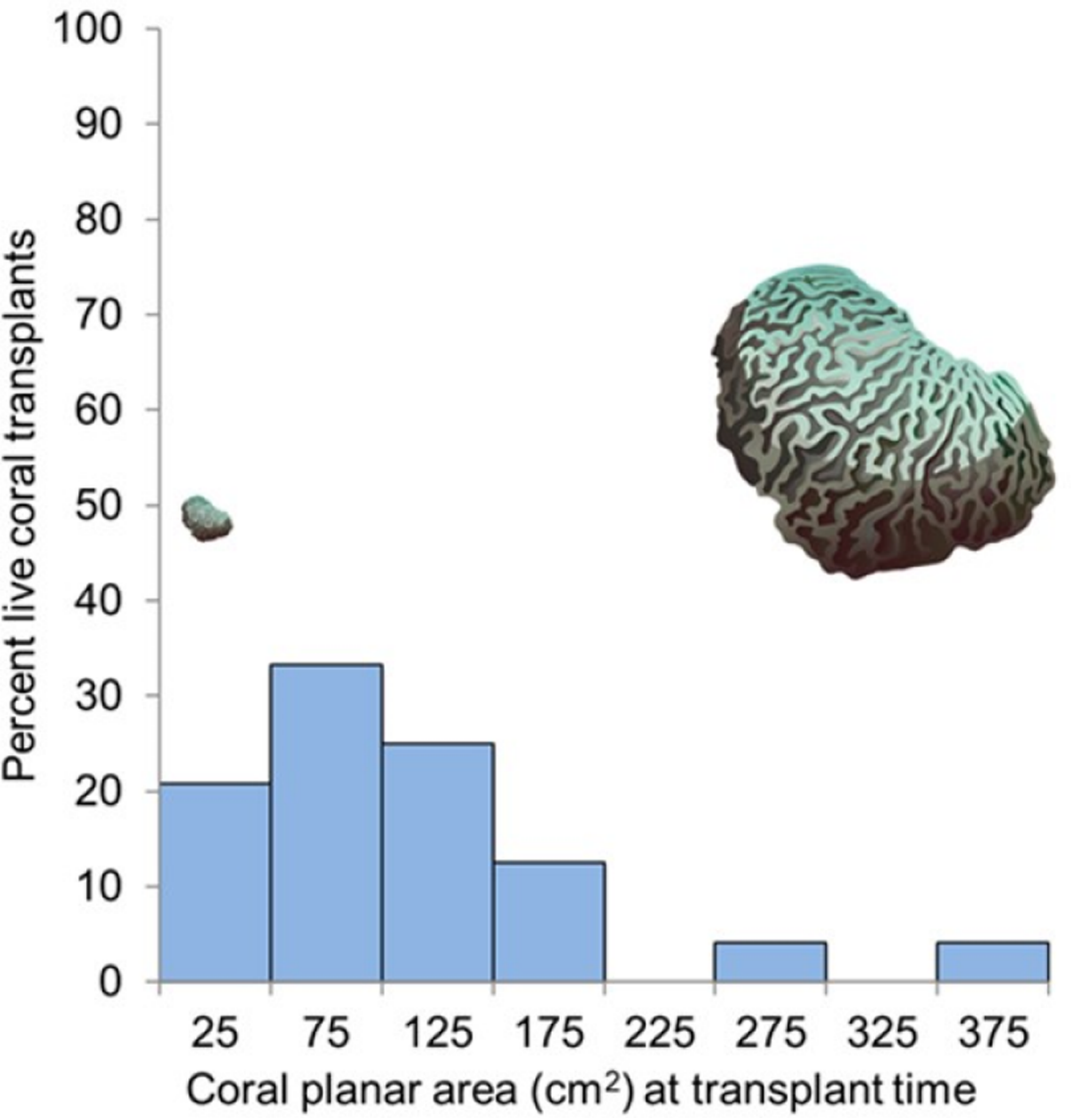
Coral survival related to size at transplant. Percentage of corals surviving 11.5 months after transplantation as it relates to their size at transplant for the 48 nursery-grown corals monitored in this study. The coral images show relative sizes for visual comparison. Credit for coral symbols: Woerner (2011).

The average coral planar area at transplant time was 117.4 cm^2^ (SE 11.57), range 30.7 to 384.5 cm^2^. Survival 11.5 months post-transplantation did not increase with size at transplant (two-tailed *t*-test; *t* (23) = 0.607, *p* = 0.55). The overall survival rate of coral transplants at the end of the 11.5 months (345 days) experimental period ranged from 16.6% to 83.3% (branching species) and 50% to 100% (encrusting/massive species) (Fig. 4, Fig. S1).

A logistic regression analysis was used to predict survival 11.5 months post- transplantation for the 48 corals monitored using transplant zone (zones 1 through 4) and growth type (branching *vs.* massive/encrusting) as variables. A test of the full model against a constant-only model was statistically significant indicating that zone and growth type reliably distinguished between survivors and casualties (*χ*2 (2) = 7.299, *p* = 0.026)

Nagelkerke’s R^2^ of 0.19 indicated a weak relationship between prediction explained by the regression model and the grouping of variables. Prediction success overall was 68% (65% for survivors and 73% for deaths). The Wald criterion shows that only growth type made a significant contribution to prediction (*p* = 0.0102). Zone was not a significant predictor (*p* = 0.886).

### Growth of coral transplants

The overall mean change in colony size (CCS) was 10.1 cm^2^ ± 8.8 (SE), range −128.5 to 175.9 cm^2^, and was normally distributed (Shapiro–Wilk normality test *W* = 0.9711, *p* = 0.18). The lowest range value was a branching coral in Zone 3 with an 83% reduction in colony size due to mortality. The highest range value was a branching coral in Zone 4 with a 51% increase in colony size.

The annual extension rate (mm/y) calculated by geometry, had positive (growth) and negative (partial mortality) values. When growth occurred, it was within the range published in the literature for 3 branching species (*Pocillopora grandis*, *P. damicornis*, *P. verrucosa*) and 1 massive species (*Astreopora myriophthalma*), but 1 submassive to encrusting species (*Favites cf pentagona*) had both higher and lower growth rates than previously published (Table S1).

A single sample *t*-test revealed that the mean CCS was not significantly different from 0 (t (57) = 1.1552, *p* = 0.2528, 95% confidence interval of the mean = [−6.9–28.2] after 10 000 bootstrapped samples). Thus, our data suggest that there was no significant change in colony size one year after transplantation.

**Figure 5:**
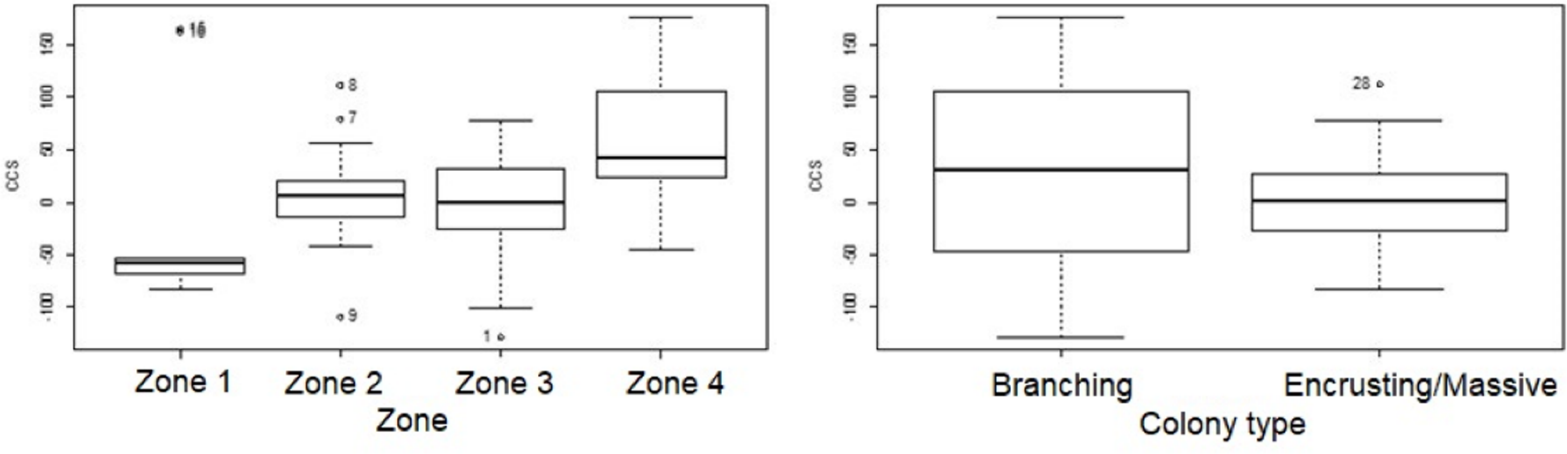
Coral transplant growth related separately to restoration zone and growth type. Coral transplant growth, defined as net change of colony size (CCS), related to restoration zone (left) and growth type (right). The variation within zones was not homogeneous (Bartlett’s K-squared = 8.2871, *df* = 3,* p* = 0.04044). The variation within growth types was not homogeneous (Bartlett’s K-squared = 17.5512, *df* = 1, *p* = 2.797e−05).

**Figure 6:**
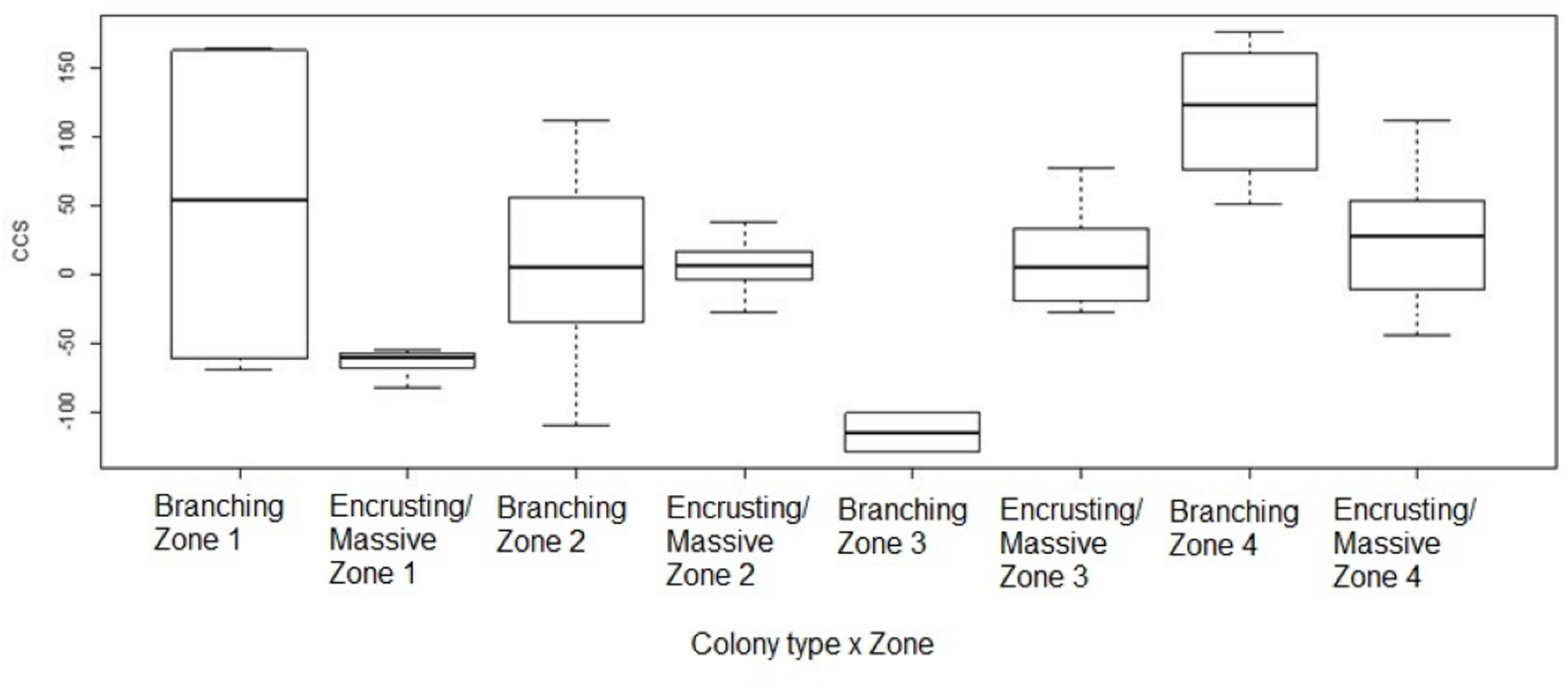
Coral transplant growth related to both restoration zone and growth type. Coral transplant growth, defined as the net change of colony size (CCS), with the combined effects of restoration zone and growth type. Overall CCS for branching colonies was higher than for massive/encrusting colonies (GLMM, *ζ* = 2.78, *p* = 0.005) and varied significantly among zones.

**Figure 7:**
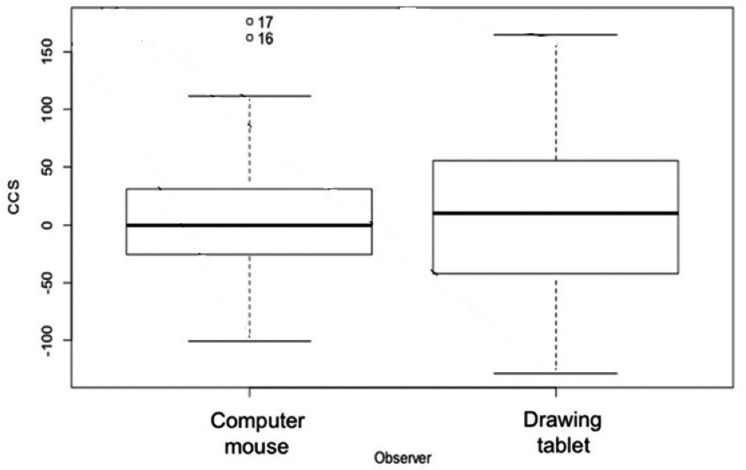
Coral transplant growth measured in ImageJ by two different methods. Coral transplant growth, defined as the net change of colony size (CCS), when comparing the two measuring methods (computer mouse *vs.* drawing tablet). The variation within observers was homogeneous (Bartlett’s K-squared = 0.5574, *df* = 1, *p* = 0.4553).

Change in colony size (Figs. 5 and 6) varied between zones (Zone 1: −17.9 cm^2^ ± 30.3 *n* = 10; Zone 2: 7.7 cm^2^ ± 9.6 *n* = 22; Zone 3: −6.0 cm^2^ ± 14.9 *n* = 14; Zone 4: 56.8 cm^2^ ± 19.7 *n* = 12) and growth types (Branching: 27.3 cm^2^ ± 21.6 *n* = 20; Massive/Encrusting: 1.1 cm^2^ ± 6.9 *n* = 38). The Likelihood ratio tests showed a significant interaction effect between zone and growth type on the average change in colony size (*χ*2 (3) = 13.9, *p* < 0.003; Table S2). The effect of each colony was substantial (ca. 99%). The estimated variance for Observer as a random effect was virtually zero suggesting no significant effect between independent observers when measuring CCS (Table S3, Fig. 7).

Overall CCS for branching colonies was higher than for massive/encrusting colonies (GLMM, *z* = 2.78, *p* = 0.005) and varied significantly among zones (Table S3), increasing from Zone 1 to Zone 4. Monitored colonies from Zone 1 showed higher levels of partial mortality (*i.e.,* lower CCS values) compared to colonies from other zones. Coral in Zone 4 grew more than corals in the other three zones (GLMM, *z* = 2.60, *p* = 0.009; Table 2) and the mean CCS difference between Zone 4 and Zone 1 was 89.9 cm^2^. Branching colonies in Zone 3 had significantly lower CCS suggesting higher partial mortalities (GLMM, *z* = 3.78, *p* < 0.001; Table S3).

## Discussion

The boutique restoration approach was feasible. The site selection, coral species selection, and implementation of restoration activities complied with the principles of ecological restoration (Suding et al., 2015) while at the same time satisfying the needs of the hotel resort.

Although we developed a simple monitoring protocol and trained hotel staff to monitor coral transplants monthly, high hotel personnel turnover and lack of allocated time prevented trained staff to implement monitoring with the frequency required to complete standard survival analysis. Such loss of capacity building to work and monitor the restoration activity is a major setback even in the best-planned restoration programs (Lirman & Schopmeyer, 2016). In the context of private sector engagement, hotel resorts have been recognized as instrumental private sector partners in coral reef restoration (Rinkevich, 2015). But to fully participate as partners, hotel resorts must commit to all phases of the restoration activity, including training the hotel staff and protecting the time needed for monitoring restoration success. Large hotel networks are starting a strategy of increasing their engagement in coral reef restoration at different scales (Bayraktarov et al., 2020), even with dedicated staff and resources allocated to restoration and monitoring (Iberostar Hotels and Resorts, Blanco-Pimentel et al., 2022). In this way, boutique restoration activities where scientists and hotel staff work together could be a localized solution to coral reef restoration. Unless we develop socio-economic strategies that benefit local communities, with the involvement of the public and private sectors, coral reef restoration will continue to rely on short-term grants and donations, and the ability to implement comprehensive restoration strategies will be limited (Frias-Torres et al., 2021).

The simplified monitoring method was robust enough to detect the expected performance differences in coral transplants, with higher survival in massive and encrusting coral transplants when compared to branching coral transplants (Seguin et al., 2008; Gomez et al., 2014). Survival of encrusting and massive corals was higher (50%–100%) than branching corals (16.6%–83.3%). Growth type rather than transplant zone was a better predictor of coral transplant survival. There was no size refuge for corals, and coral survival did not increase for corals with a larger planar area at transplant time, even when the largest size class was 15 times larger than the smallest size class. Other studies have shown large coral transplants with a surface area of roughly 1,000 cm^2^, have higher survival than small (about 10 cm^2^) and medium-sized corals (about 100 cm^2^) (Forrester et al., 2014). Since the planar area of our coral transplants ranged from 30.7 to 384.5 cm^2^, we were unable to detect differences in survival related to a size range extending through two orders of magnitude.

The overall change in colony size one year after transplantation was non-significant, although the positive mean suggests there was some colony growth. The non-significant change in colony size is most likely explained by the length of the study. Once transplanted, colonies need to invest more energy in attaching and adjusting to the new environmental conditions which will result in low or no growth at all (Yap, Aliño & Gomez, 1992). An extensive review of growth values obtained through density banding patterns, alizarin red staining, direct tagging, photographic analysis, or a combination of these techniques (Pratchett et al., 2015) shows the corals monitored in this study, when comparisons were possible, had similar growth rates for two branching species (*Pocillopora grandis*, *Pocillopora damicornis*) and one encrusting/massive species (*Astreopora myriophthalma*). However, growth rates were lower than the reference in three branching species (*Acropora humilis*, *Acropora abrotanoides*, *Pocillopora verrucosa*) and five encrusting/massive species (*Favites cf flexulosa*, *Favites cf pentagona*, *Dipsastrea cf favus*, *Astreopora myriophthalma*, *Platygyra cf crosslandi*) (Table S1).

The distinct individual differences in growth, regardless of transplant zone or growth type, explained the variations in change in colony size (CCS). Although monitored colonies were grouped by growth type, branching *vs.* encrusting/massive, each growth type was represented by more than one species. Such growth variation highlights the species-specific response to the effects of transplantation on colony growth (Yap, Aliño & Gomez, 1992; Yap et al., 1998). Some growth values were negative. This is due to the intrinsic nature of growth in colonial organisms such as corals. A proportion of polyps within the coral colony can die, yet the coral colony is still alive. This partial mortality means that coral colonies can decrease (negative growth) as well as increase (positive growth) in size (Pratchett et al., 2015).

There was a significant zone effect on both the growth and survival of coral transplants. The zoning effect on growth was detected as an overall mean CCS increase from Zone 1 to Zone 4, suggesting a corresponding decrease in partial coral transplant mortality. This differential change in colony size can be explained by the level of exposure in each zone. Zone 1 and Zone 4 were the most exposed zones to waves and sediment load, and the predominant exposure alternated between the Southeast and Norwest Monsoon seasons. Zone 4 was better protected by the granite boulders of the area. Zone 2 and Zone 3 were the most protected from wave exposure and associated sediment load. The zoning effect on survival was linked to growth type. For encrusting/massive corals Zone 2 and Zone 3 had the highest survival (100% in both zones) followed by Zone 4 and Zone 1 (66.7% and 50% survival respectively). In contrast, for branching corals, Zone 2 had the highest survival (83.3%) followed by Zone 1, Zone 4, and Zone 3 (33.3%, 33.3%, and 16.7% survival respectively). In coral atolls, wave forcing and exposure structured coral reef benthic community composition and recovery trajectories after a bleaching event. Recovery trajectories at sheltered and exposed sites were similar showing that in the absence of human stressors, community patterns on fore reefs are strongly controlled by wave exposure, even during and after widespread coral loss from bleaching events (Lange et al., 2021). A meta-analysis of a global dataset for over 140 coral species throughout the Atlantic, Indian, and Pacific oceans about the effects of sediment exposure on corals showed that there is enormous variation in the levels of exposure to deposited and suspended sediment that corals can tolerate. Adverse effects, including mortality, occur at deposited sediment concentrations as low as 1 mg/cm^2^/day and occur within hours to days, but within days to weeks for suspended sediment concentrations as low as 3.2 mg/L. There is limited evidence that coral adults are less sensitive than immature stages to either deposited or suspended sediment (Tuttle & Donahue, 2022).

Branching coral growth is measured as a branch or vertical extension but encrusting and massive coral growth is measured as a horizontal extension (Pratchett et al., 2015). We were limited by the need to use a fast and simple measuring technique, to overcome the challenges of measuring during heavy swells at shallow depths, and with a protocol that required minimal training so it could be implemented by non-expert hotel staff in the future. Branching corals grow both in height and width, and this was detected by the coral planar area measurement. The use of a standard computer mouse or a stylus with a tablet had no significant differences when tracing the coral planar area in Image J. Therefore, the requirements needed to follow our monitoring protocol do not exceed the standard hardware and software capabilities of a hotel resort.

We limited monitoring efforts to one-year (11.5 months) post-transplantation. This initial period has the highest mortality, perhaps reflecting stress from handling or failure during the attachment period (Forrester et al., 2014). We are aware that longer monitoring periods can offer a more realistic view of the restoration response (Montoya-Maya et al., 2016).

A comprehensive approach to the boutique restoration monitoring experiment would have included comparisons with a control patch reef with a similar species composition to the coral transplants. However, the ability to monitor such a control site, in addition to the restoration site, was beyond the logistic capabilities and resources of the hotel staff, and we were limited to monitoring survival and growth within the boutique restoration site. We had a historical baseline for Petite Anse Kerlan. Before the mass coral mortality from the 1998 El Nino and Indian Ocean Dipole coupling, the exposed sandy bay of Petite Anse Kerlan contained a diverse shallow-water coral reef (N. Shah pers. comm., 2014), with “old coral blocks, and large rock formations” (Van der Land, 1994) (Appendix S1 -Methods). Using the four principles of ecological restoration (Table 1, Suding et al., 2015) we replicated as much as possible the restoration of the coral growth forms found at the site before the mass coral mortality event.

For future work, we recommend including two control sites, one degraded site without restoration, and a second site with a similar species composition to the restoration site. Survival and growth results can be compared then between the sites (Frias-Torres et al., 2021). The low-tech monitoring method reported here could be extended with additional information before or after taking the photographs, including the use of visual tools to monitor bleaching, disease, and predators. We published detailed monitoring protocols for coral reef restoration elsewhere (Frias-Torres, Montoya-Maya & Shah, 2019).

Finally, recent advances in the restoration of massive and encrusting corals allow for rapid production of clones using micro-fragmentation where small (∼1 cm^2^) micro-fragments are cut from the same colony, spaced regularly over ceramic tiles, and spread at rapid rates (*e.g.*, tens of square centimeters per month) followed by tissue fusion (Forsman et al., 2015). Micro-fragmentation can also eliminate the nursery phase for massive and encrusting corals with direct transplantation to the substrate after fragmentation. For example, in the Caribbean mountainous star coral, *Orbicella faveolata*, micro-fragments produced 10 times more tissue than traditionally used larger fragments 31 months post-transplantation (Page, Muller & Vaughan, 2018). When we completed the boutique restoration project, the knowledge and techniques required for micro-fragmentation were not available to us. In future projects, we recommend using both the coral gardening method with branching and tabular corals, to provide immediate three-dimensional structure at transplantation time, combined with micro-fragmentation and fusion to ensure fast growth for massive and encrusting coral transplants.

## Conclusions

Our low-tech monitoring method was robust enough to detect expected survival differences between coral transplants in a very shallow patch reef, with higher survival in massive and encrusting corals compared to branching corals.

Although we trained hotel staff successfully in this method, high hotel personnel turnover and lack of allocated time prevented trained staff to implement monthly monitoring to complete standard survival analysis. We were only capable of monitoring coral transplant survival and growth at the start of the experimental period and 11.5 months post-transplantation.

We conclude that science-based boutique coral reef restoration, tailored to the needs of a hotel resort, combined with the capability of hotel staff to implement a simple monitoring method and evaluate restoration success, could provide a framework for involving hotels as partners in coral reef restoration worldwide. To fully participate as partners, hotel resorts must commit to all phases of the restoration activity, including training of hotel staff and protecting the time needed for monitoring restoration success.

Our study fills a gap in the need for rapid and simple methods, available to hotel resort staff, for monitoring coral reef restoration. This is a critical first step in building a framework for involving the hotel resort private sector as partners in coral reef restoration worldwide.

##  Supplemental Information

10.7717/peerj.15062/supp-1 Supplemental Information 1 Appendix, Supplemental Tables, Figure

10.7717/peerj.15062/supp-2 Supplemental Information 2 Image J measurements Raw data and analysis of Image J measurements, survival and growth of coral transplants.

## Additional Information and Declarations

### Data Availability

The following information was supplied regarding data availability: The raw data are available in the Supplemental Files.
