# Supplementary material for: A low-tech method for monitoring survival and growth of coral transplants at a boutique restoration site
Source: PeerJ. 2023 May 24;11:e15062. doi: 10.7717/peerj.15062 (PMC10224673; doi:10.7717/peerj.15062)

**SUPPORTING INFORMATION**

**Appendix S1-Methods**

***Study site description***

The boutique restoration experiment was conducted at Petite Anse Kerlan, Praslin Island (04˚17'57.5'' S, 055˚40'40.0'' E WGS84) (Fig 1). The site is semi-protected from prevailing winds due to large granite boulders around the bay and a rock island at the entrance of the bay. The shallow (1-3 m deep) site is influenced by tides, ocean swells, and salinity fluctuations linked to the Monsoons. From May to October, the trade winds blow from the southeast (Southeast Monsoon), and from December to March they blow from the northwest (Northwest Monsoon). The transition months of April and November have light and variable winds

Before the mass coral mortality from the 1998 El Nino and Indian Ocean Dipole coupling, the exposed sandy bay of Petite Anse Kerlan contained a diverse shallow-water coral reef (N. Shah personal communication), with "old coral blocks, and large rock formations" (van der Land 1994). Since the 1998 mass bleaching event, coral rubble deposits had accumulated underwater on the northeastern edge of the bay, mostly stabilized by coralline algal growth. The shallow central area of the bay consists of soft, fine sediment transitioning into sparse macroalgal growth at the exposed rocky substrate.

***Coral growth types***

We used Veron (2000) to define growth types: branching corals form branches in complex three-dimensional habitat, massive corals are solid and ball or boulder shaped, and encrusting corals cover the substrate in a sheet expanding in diameter rather than height. When more than one growth type was possible for a species, we classified it based on what we grew at the net nurseries.

***Cementing corals to a hard substrate***

We cemented corals onto the granite and limestone substrate with a unique mix developed by one of us (C. Reveret). Briefly, Portland marine cement was mixed with Sikacrete ® UCS, a stabilizer powder that increases cohesion and reduces washout when cement is applied underwater. The Sikacrete® to marine cement ratio used is proportional to the expected wave energy at the transplantation site. In a large-scale coral reef restoration project at nearby Cousin Island, where the 12 m deep transplantation site had some swell, we used a cement mix with 3 % sikacrete (Frias-Torres et al. 2018). At the Petite Anse Kerlan transplantation site, the shallow site was highly influenced by wave energy. At this site, we used a cement mix with 10 % Sikacrete®. Once the marine cement and Sikacrete® was thoroughly mixed in dry form, freshwater was added slowly and the mix was worked by hand similar to kneading dough until a dough-like consistency was reached. We then filled 40 cm long nylon pastry bags. The bag tip was cut and held closed with a clothespin. The back of the bag was closed with rope. The pastry bag facilitated accurate injection of cement to secure the coral to the substrate with minimal waste of the cement mix. This was done by squeezing and twisting the pastry bag from the back like a tube of toothpaste (Fig. 2).

**Literature cited**

Van der Land J (1994) The “Oceanic Reefs” Expedition to the Seychelles. Zool. Verh. Leiden 297: 5-36

Veron JEN (2000) Corals of the World. Australian Institute of Marine Science, Townsville, Australia.

**Table S1** **List of species monitored.** Published growth values obtained through density banding patterns, alizarin red staining, direct tagging, photographic analysis, or a combination of these techniques (Pratchett et al. 2015) and those calculated in this study are shown. Legend: n/a, data not available; (*) data only available for the genus.

| Growth type | Coral Code | **Species (pka = previously known as)** | | **Annual extension rate**  **(mm yr^-1^)** | |
| --- | --- | --- | --- | --- | --- |
| Zone 1 | | | Pratchett et al (2015) | | This study |
| Branching | Z1B1 | *Acropora abrotanoides (*pka. *Acropora irregularis)* | 125 - 130 | | n/a |
|  | Z1B2 | *Acropora cf cytherea* | 20.9 – 93.2 | | n/a |
|  | Z1B3 | *Acropora humilis* | 9.6 – 23.7 | | -32.0 |
|  | Z1B4 | *Acropora abrotanoides (*pka. *Acropora irregularis)* | 125 - 130 | | n/a |
|  | Z1B5 | *Pocillopora grandis (*pka*. Pocillopora eydouxi)* | 20 – 50.4 | | 29.9 |
|  | Z1B6 | *Acropora muricata (*pka. *Acropora formosa)* | 58.5 –120.9 | | n/a |
| Encrusting/  Massive | Z1E1 | *Favites cf flexulosa* | 9.6 * | | -20.6 |
|  | Z1E2 | *Echinopora hirsutissima* | n/a | | n/a |
|  | Z1E3 | *Favites cf flexulosa* | 9.6 * | | n/a |
|  | Z1E4 | *Coscinarea monile* | n/a | | n/a |
|  | Z1E5 | *Favites cf pentagona* | 9.6 * | | -34.7 |
|  | Z1E6 | *Echinopora hirsutissima* | n/a | | -15.9 |
| Zone 2 | | |  | |  |
| Branching | Z2B1 | *Pocillopora damicornis* | 6.6 – 66.8 | | -10.7 |
|  | Z2B2 | *Isopora brueggemanni (*pka. *Acropora brueggemanni)* | n/a | | n/a |
|  | Z2B3 | *Pocillopora damicornis* | 6.6 – 66.8 | | 18.8 |
|  | Z2B4 | *Acropora abrotanoides (*pka*. Acropora irregularis)* | 125 - 130 | | -18.3 |
|  | Z2B5 | *Pocillopora verrucosa* | 17.8 – 37.2 | | -1.9 |
|  | Z2B6 | *Pocillopora verrucosa* | 17.8 – 37.2 | | 12.8 |
| Encrusting/  Massive | Z2E1 | *Favites cf pentagona* | 9.6 * | | 4.0 |
|  | Z2E2 | *Dipsastrea cf favus (*pka. *Favia cf favus)* | 2.8 –12.7** | | -1.2 |
|  | Z2E3 | *Astreopora myriophthalma* | 7.5 - 13 | | 5.1 |
|  | Z2E4 | *Astreopora myriophthalma* | 7.5 - 13 | | 8.5 |
|  | Z2E5 | *Astreopora myriophthalma* | 7.5 - 13 | | -6.0 |
|  | Z2E6 | *Echinopora hirsutissima* | n/a | | 0.3 |
| Zone 3 | | |  | |  |
| Branching | Z3B1 | *Acropora cf verweyi* | n/a | | -36.7 |
|  | Z3B2 | *Acropora humilis* | 9.6 – 23.7 | | n/a |
|  | Z3B3 | *Acropora cf verweyi* | n/a | | n/a |
|  | Z3B4 | *Acropora humilis* | 9.6 – 23.7 | | n/a |
|  | Z3B5 | *Acropora abrotanoides (*pka. *Acropora irregularis)* | 125 - 130 | | n/a |
|  | Z3B6 | *Pocillopora grandis (*pka. *Pocillopora eydouxi)* | 20 – 50.4 | | n/a |
| Encrusting/  Massive | Z3E1 | *Favites cf pentagona* | 9.6 * | | 1.8 |
|  | Z3E2 | *Goniopora pedunculata (*pka*. Goniopora minor)* | n/a | | 5.4 |
|  | Z3E3 | *Echinopora hirsutissima* | n/a | | -6.8 |
|  | Z3E4 | *Favites cf pentagona* | 9.6 * | | 15.3 |
|  | Z3E5 | *Favites cf pentagona* | 9.6 * | | 17.3 |
|  | Z3E6 | *Platygyra cf crosslandi* | 4.9 – 12 * | | -7.7 |
| Zone 4 | | |  | |  |
| Branching | Z4B1 | *Acropora cf abrotanoides (*pka. *Acropora irregularis)* | 125 - 130 | | n/a |
|  | Z4B2 | *Acropora cf abrotanoides (*pka*. Acropora irregularis)* | 125 - 130 | | n/a |
|  | Z4B3 | *Acropora cf verweyi* | n/a | | n/a |
|  | Z4B4 | *Pocillopora grandis (*pka. *Pocillopora eydouxi)* | 20 – 50.4 | | 12.2 |
|  | Z4B5 | *Pocillopora grandis* (pka. *Pocillopora eydouxi)* | 20 – 50.4 | | 21.2 |
|  | Z4B6 | *Acropora cf verweyi* | n/a | | n/a |
| Encrusting/  Massive | Z4E1 | *Galaxea fascicularis* | n/a | | 15.1 |
|  | Z4E2 | *Echinopora hirsutissima* | n/a | | 10.2 |
|  | Z4E3 | *Echinopora hirsutissima* | n/a | | n/a |
|  | Z4E4 | *Favites cf pentagona* | 9.6 * | | n/a |
|  | Z4E5 | *Favites cf flexulosa* | 9.6 * | | -15.5 |
|  | Z4E6 | *Hydnophora exesa* | n/a | | 12.4 |

**Literature cited**

Pratchett MS, Anderson KD, Hoogenboom MO, Widman E, Baird AH, Pandolfi JM, Edmunds PJ, Lough JM (2015) Spatial-temporal and taxonomic variation in coral growth: Implications for the structure and function of coral reef ecosystems. Oceanography and Marine Biology: An Annual Review 53: 215-295.

**Table S-2** **Summary of statistics.** Summary of generalized linear mixed-effect model comparisons using Likelihood ratio test. AIC = Akaike’s Information Criterion; BIC = Bayesian Information Criterion.

*Models:*

*Model_null: CCS ~ 1 + (1 + 1 | Coral_code) + (1 + 1 | Observer)*

*Model_1: CCS ~ Colony_type + (1 + 1 | Coral_code) + (1 + 1 | Observer)*

*Model_2: CCS ~ Zone + (1 + 1 | Coral_code) + (1 + 1 | Observer)*

*Model_3: CCS ~ Zone + Colony_type + (1 + 1 | Coral_code) + (1 + 1 | Observer)*

*Model_full: CCS ~ Zone * Colony_type + (1 + 1 | Coral_code) + (1 + 1 | Observer)*

*Df AIC BIC logLik deviance Chisq Chi Df Pr(>Chisq)*

*Model_null 4 618.53 626.77 -305.26 610.53*

*Model_1 5 619.41 629.72 -304.71 609.41 1.1143 1 0.291148*

*Model_2 7 619.51 633.93 -302.75 605.51 3.9040 2 0.141991*

*Model_3 8 620.23 636.72 -302.12 604.23 1.2776 1 0.258344*

*Model_full 11 612.30 634.96 -295.15 590.30 13.9353 3 0.002995 ***

*---*

*Signif. codes: 0 '***' 0.001 '**' 0.01 '*' 0.05 '.' 0.1 ' ' 1*

**Table S-3** **Effects of zone and growth type on CCS**. Results of GLMM of the effects of Zone and Growth type on CCS. Zone 1 and Encrusting were set as the reference levels when building the GLMM.

*Model = CCS ~ Zone * Colony_type + (1 + 1 | Coral_code) + (1 + 1 | Observer), data = CCS, family = "Gaussian")*

*AIC: 612.3*

*Coefficients:*

*Estimate Std. Error z value Pr(>|z|)*

*(Intercept) -63.7 26.1 -2.44 0.01463 **

*Zone 2 69.6 32.0 2.18 0.02950 **

*Zone 3 75.8 32.0 2.37 0.01772 **

*Zone 4 89.9 34.5 2.60 0.00920 ***

*Colony type Branching 114.6 41.3 2.78 0.00548 ***

*Zone 2: Branching -110.5 49.5 -2.23 0.02558 **

*Zone 3:Branching -241.4 63.9 -3.78 0.00016 ****

*Zone 4:Branching -22.6 56.9 -0.40 0.69062-*

*Signif. codes: 0 '***' 0.001 '**' 0.01 '*' 0.05 '.' 0.1 ' ' 1*

*Number of observations: total=58, Coral_code=29, Observer=2*

*Random effect variance(s):*

*Group=Coral_code*

*Variance StdDev*

*(Intercept) 1753 41.87*

*Group=Observer*

*Variance StdDev*

*(Intercept) 7.172e-09 8.469e-05*

*Residual variance: 24.131 (std. err.: 3.1681)*

*Log-likelihood: -295.149*

**Figure S1** **Survival related to zone and growth type.** Survival related to restoration zone and growth type for the 48 corals monitored.


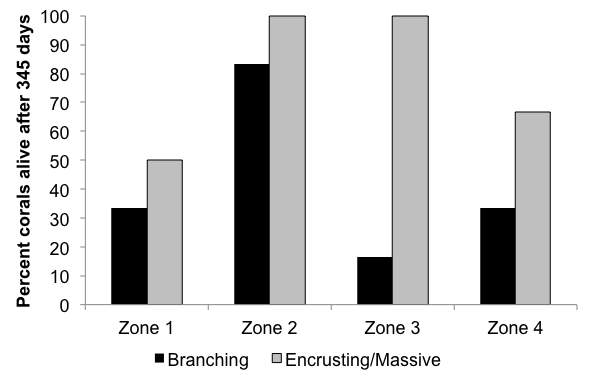

Supplement: Supplemental Information 1 [file peerj-11-15062-s001.docx]
